# Supplementary material for: Sour Beer as Bioreservoir of Novel Craft Ale Yeast Cultures
Source: Microorganisms. 2023 Aug 23;11(9):2138. doi: 10.3390/microorganisms11092138 (PMC10537276; doi:10.3390/microorganisms11092138)
Supplement: Supplementary file 1 [file microorganisms-11-02138-s001.zip › microorganisms-2528837-supplementary.pdf]

Article

Sour beer as bioreservoir of novel craft ale yeast cultures

Chiara Nasuti [1], Jennifer Ruffini [1], Laura Sola [2], Mario Di Bacco [3], Stefano Raimondi [2], Francesco Candeliere [2], Lisa Solieri [1,4,*]

1 Department of Life Sciences, University of Modena and Reggio Emilia, Via Amendola, 2-Pad. Besta, 42122 Reggio Emilia, Italy; chiara.nasuti@unimore.it (C.N.); 227818@studenti.unimore.it (J.R.)

2 Department of Life Sciences, University of Modena and Reggio Emilia, Via Campi 51, 41125 Modena, Italy; laura.sola@unimore.it (L.S.); stefano.raimondi@unimore.it (S.R.); francesco.candeliere@unimore.it (F.C.)

3 Ca' Del Brado Brewery, Via Andrea Costa, 146/2, 40065 Rastignano, Italy; quark@cadelbrado.it

4 National Biodiversity Future Center (NBFC), 90133 Palermo, Italy

* Correspondence: L.S.: lisa.solieri@unimore.it; Tel.: +39 0522522026

Supplementary Data

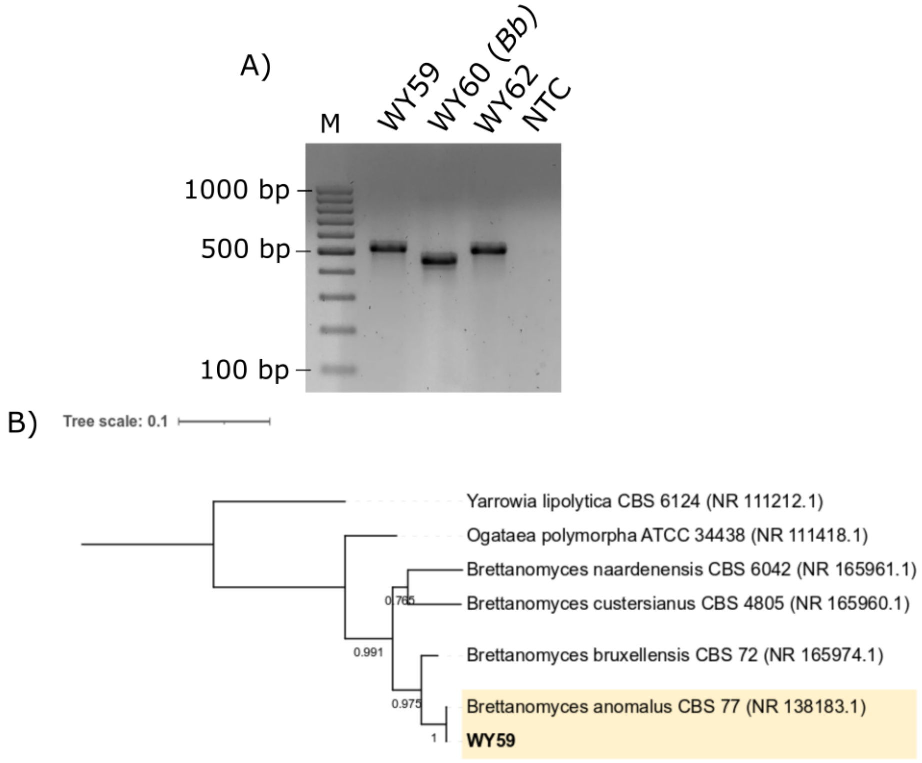

Supplementary Figure S1. Analysis of ITS region in *D. anomala* isolates. (A) Electrophoretic gel of ITS amplicons. *D. bruxellensis* strain WY60 was used as reference strain. (B) Phylogenetic tree obtained by neighbor joining (NJ) method [1] applied to a dataset of 7 rDNA sequences. The evolutionary distances were calculated by the Tamura 3-parameter method [2] considering the number of nucleotide substitutions per site. The gamma distribution was used to model the rate of change between sites. All positions containing gaps and missing data were eliminated (complete deletion option). The percentage of replicate trees in which the associated taxa clustered together in the bootstrap test [3] (1000 replicates) are shown next to the branches. The strain collected in this study is shown in bold. The lengths of the branches are proportional to the number of nucleotide substitutions, and they have been measured using the divergence scale shown at the top left. The tree was rooted using *Yarrowia lypolitica* as outgroup. The tree data (Newick) were generated with MegaX [4] and exported and visualized using ITOL [5]. Abbreviation: M: molecular size marker; Bb: *Brettanomyces bruxellensis*.

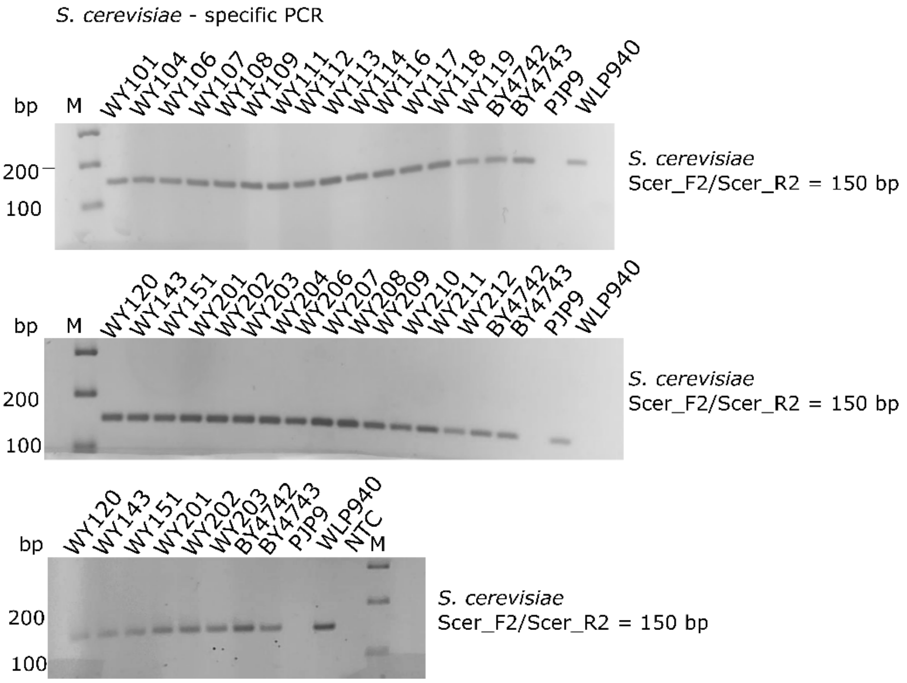

Supplementary Figure S2. *Saccharomyces cerevisiae*-specific PCRs of 35 isolates with pattern A in PCR-RFLP analysis of ITS regions. *S. cerevisiae*-specific PCR assay was carried out with Scer_F2/Scer_R2 primers targeting *MEX67* gene (amplicon size 150 bp). *S. cerevisiae* BY4742 and BY4743, *S. uvarum* PJP9, and lager strain WLP940 were used as internal control. Abbreviations: M, molecular weight marker; NTC, negative control.

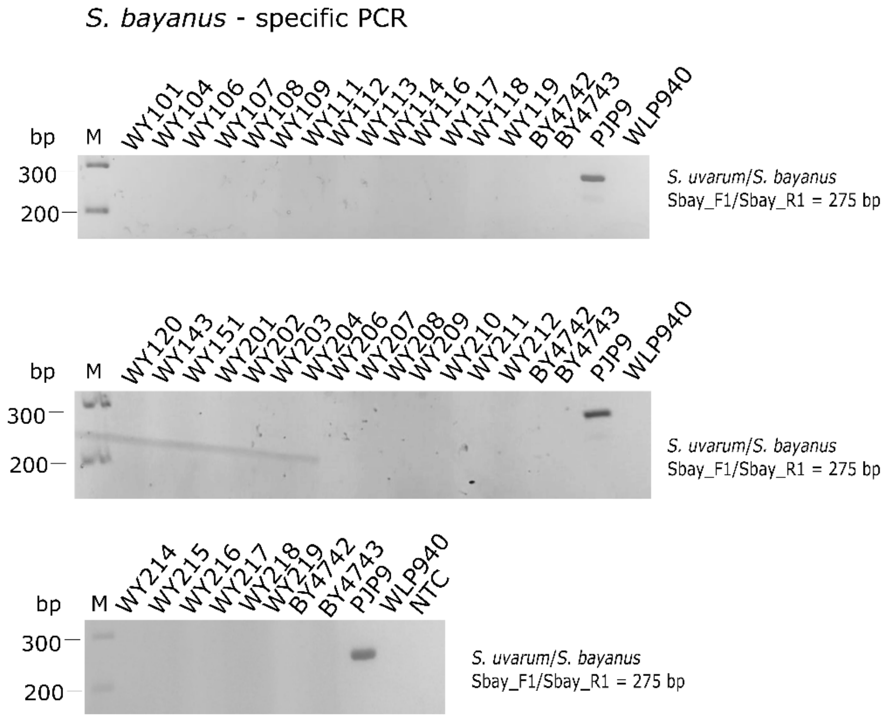

Supplementary Figure S3. *Saccharomyces bayanus*/*S. uvarum*-specific PCRs of 35 isolates with pattern A in PCR-RFLP analysis of ITS regions. *S. bayanus*-specific PCR assay was carried out with Sbay_F1/Sbay_R1 primers targeting *DBP6* gene (amplicon size 275 bp). *S. cerevisiae* BY4742 and BY4743, *S. uvarum* PJP9, and lager strain WLP940 were used as internal control. Abbreviations: M, molecular weight marker; NTC, negative control.

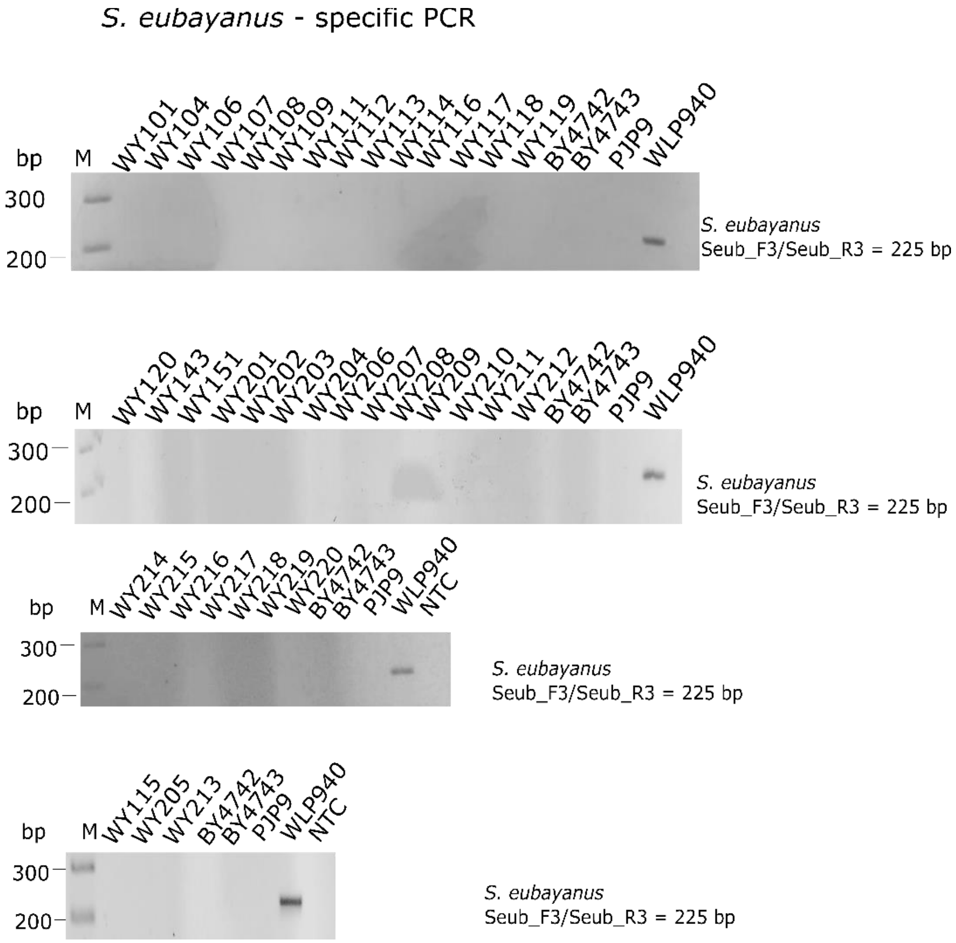

Supplementary Figure S4. *Saccharomyces eubayanus*-specific PCRs of 35 isolates with pattern A in PCR-RFLP analysis of ITS regions and of three putative *S. cerevisiae* x *S. uvarum* hybrids. *S. eubayanus*-specific PCR assay was carried out with Seub_F3/Seub_R3 primers targeting *FSY1* gene (amplicon size 228 bp). *S. cerevisiae* BY4742 and BY4743, *S. uvarum* PJP9, and lager strain WLP940 were used as internal control. Abbreviations: M, molecular weight marker; NTC, negative control.

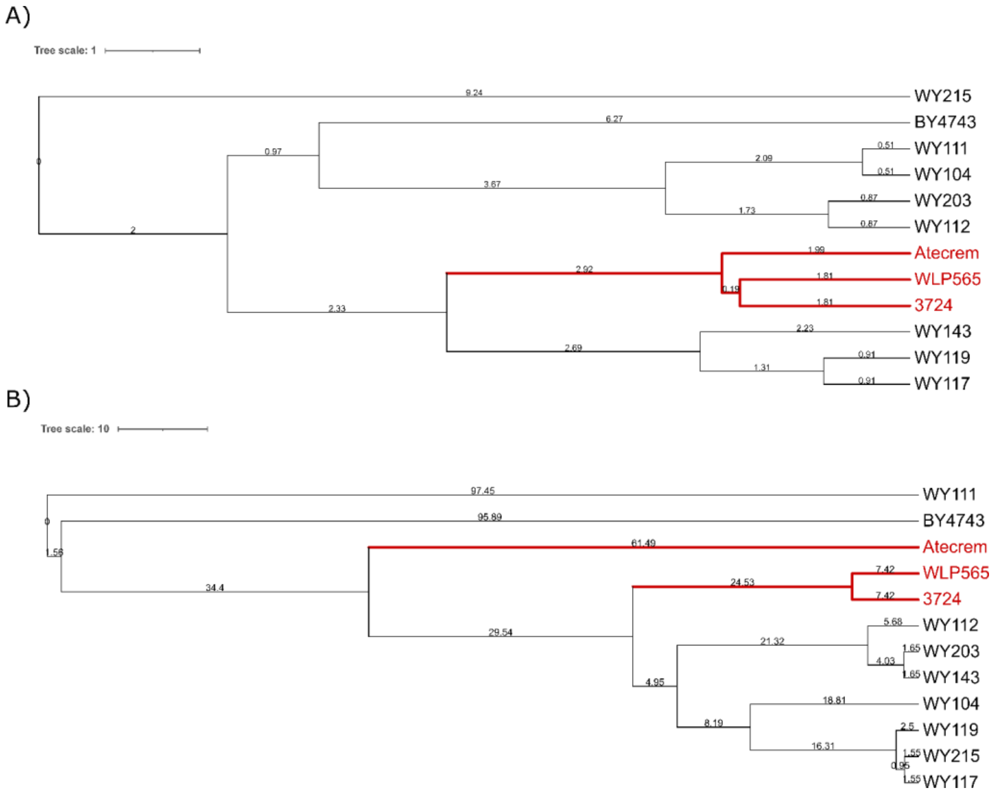

Supplementary Figure S5. Dendrograms generated using inter-delta PCR (A) and R3-RAPD PCR (B) fingerprints of 11 *S. cerevisiae* strains (7 indigenous wild strains isolated in this study from sour beer, 3 commercial starter cultures commonly used in the brewery plant, and BY4743 as reference strain). Commercial starters (in red) were detailed in Table 1. Similarity percentages were calculated using Pearson correlation coefficient, while hierarchical clustering analysis was carried out using the UPGMA (unweighted pair-group method with arithmetic mean) method with Bionumerics software. Numbers near the branches represent branch lengths. The tree data (Newick) were generated with MegaX [4] and exported and visualized using ITOL [5].

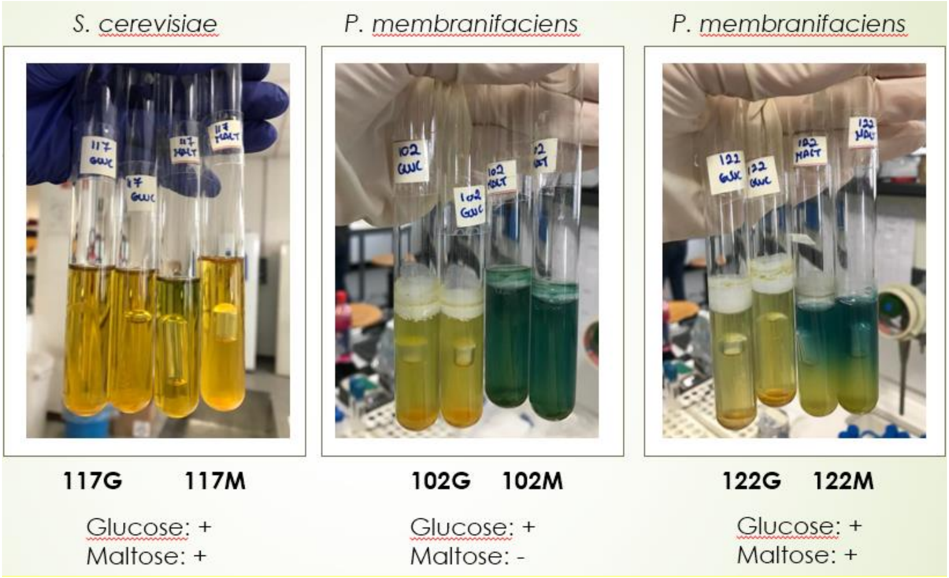

Supplementary Figure S6. Glucose and maltose fermentation test. Tubes containing Durham inverted tubes were photographed after 3 days of incubation at 27°C. Tests were carried out according to Kurtzman et al. [6]. Abbreviations: G, glucose; M, maltose.

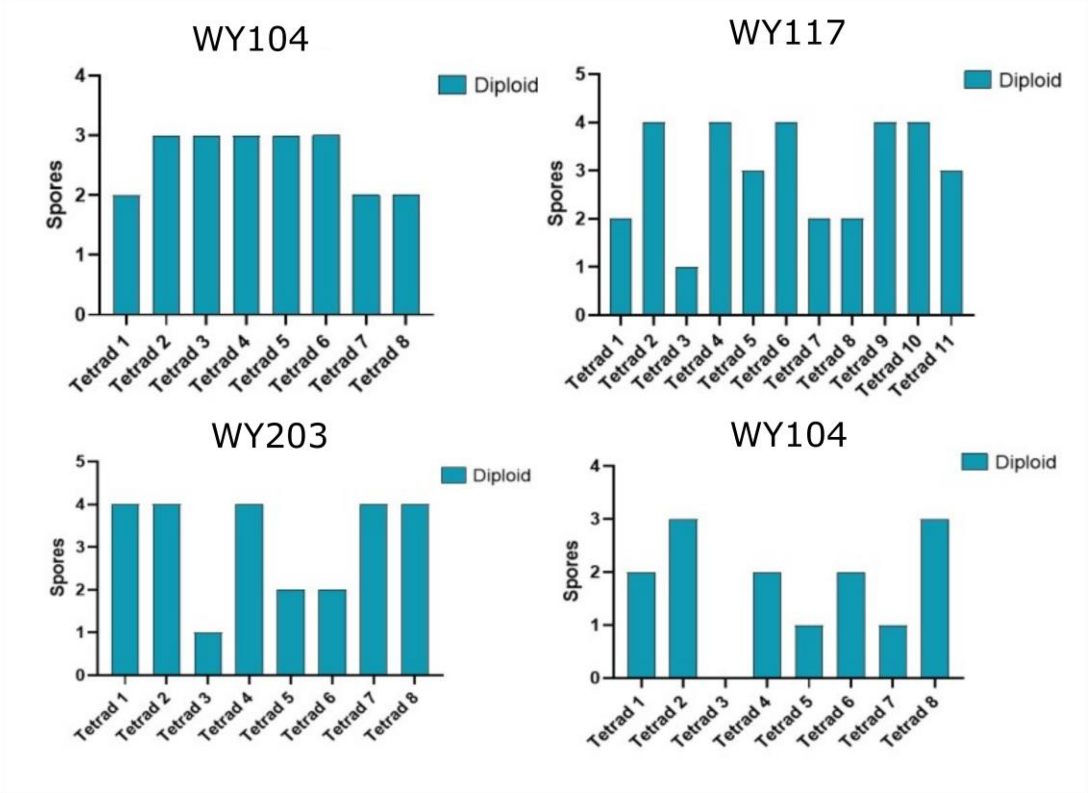

Supplementary Figure S7. Mating type genotyping of four *S. cerevisiae* sour beer wild strains and their monosporic derivatives. For each strain at least 8 meiotic events (asci) were dissected.

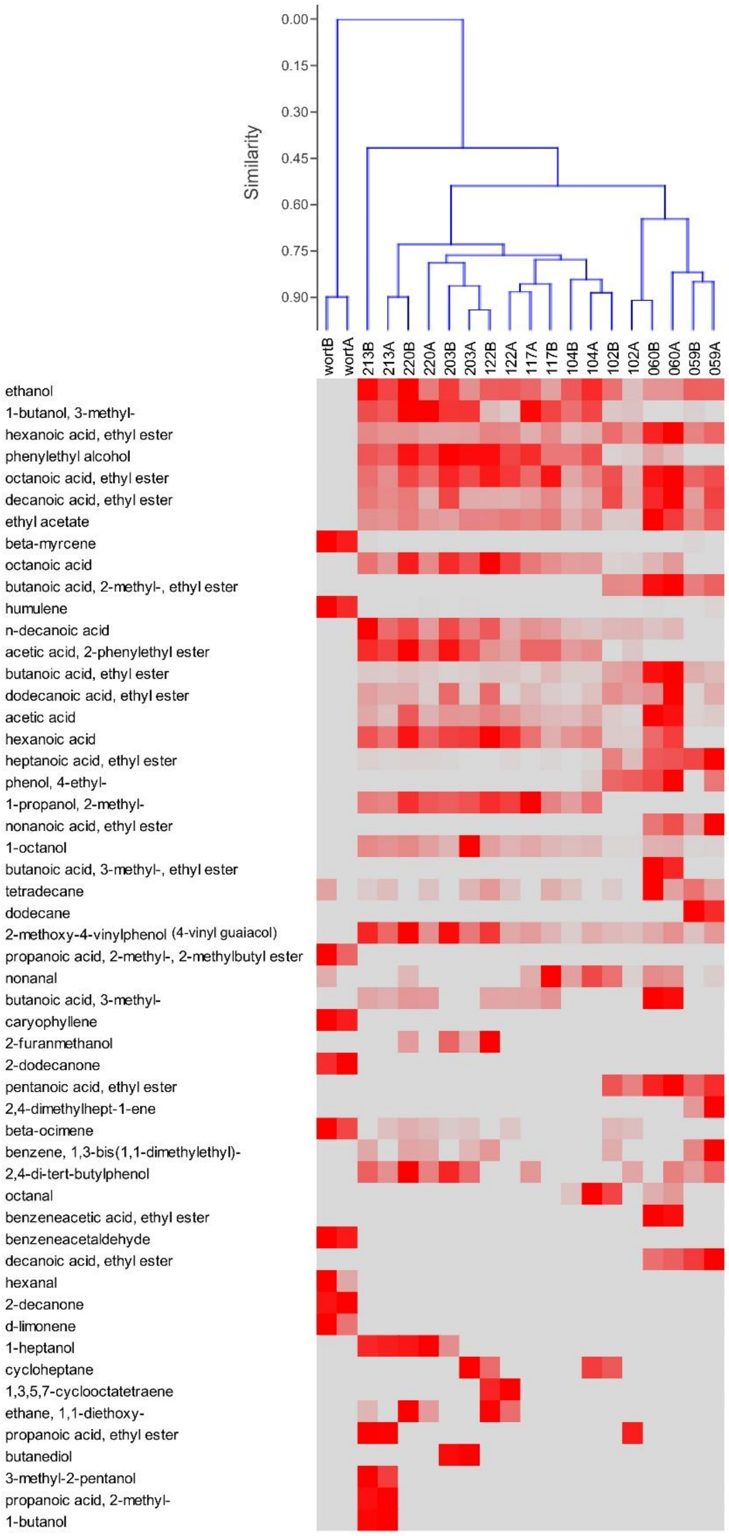

Supplementary Figure S8. Distribution of the 53 volatile organic compounds (VOCs), identified in the headspace of the fermented wort samples. To highlight the differences between samples in terms of the presence of each single VOC, rather than the relative abundance of all VOCs in a single sample, the area values of each VOC were mean centered and normalized by the corresponding standard deviation. Values are reported as colors, ranging from the lowest of each lane (grey) to the highest (deepest red).

Supplementary Table S1. Primers, compound concentrations, and thermal conditions used this study.

Goal	Gene Target	Primer name	Sequence (5′->3′)	Cycling conditions	Reaction mixture		Reference
					Vf (µL)	Compounds	
Yeast identification	ITS region	ITS1<br>ITS4	TCCGTAGGTGAACCTGCGG<br>TCCTCCGCTTATTGATATGC	95 °C for 5 min; (95 °C for 1 min, 55 °C for 2 min, 72 °C for 2 min)$_{35}$; 72 °C for 10 min		1X Dream Taq Green Buffer, 2.0 mM $MgCl_2$, 200 µM of each dNTP, 0.3 µM of each primer, 1 U of Dream Taq DNA polymerase, 100 ng DNA template	[7]
	26S LSU	NL1<br>NL4	GCATATCAATAAGCGGAGGAAAAG<br>GGTCCGTGTTTCAAGACGG-	94 °C for 5 min; 36 cycles of 94 °C for 1 min, 52 °C for 45 s, 72 °C for 2 min; final extension at 72 °C for 10 min	40	1X TaKaRa *ExTaq* Buffer*, 2.0 µM $MgCl_2$, 200 µM of each dNTP, 0.3 µM of each primer, 1 U of TaKaRa *ExTaq* DNA polymerase, and 100 ng of template DNA	[8]
	DBP6	Sbay_F1<br>Sbay_R1	GCTGACTGCTGCTGCTGCCCCCG<br>TGTTATGAGTACTTGGTTTGTCG	95°C for 3 min, (95°C for 30 sec, 58°C for 30 sec, 72°C for 1 min)35; 72°C for 7 min	20	1X Dream Taq Green Buffer, 2 mM $MgCl_2$, 200 µM of each dNTP, 0.4 µM of Sbay_F1, 0.8 µM Sbay_R1, 0.5U Dream Taq DNA polymerase, 50 ng DNA template	[9]
	MEX67	Scer_F2<br>Scer_R2	GCGCTTTACATTCAGATCCCGAG<br>TAAGTTGGTTGTCAGCAAGATTG	95°C for 3 min, (95°C for 30 sec, 58°C for 30 sec, 72°C for 1 min)$_{35}$; 72°C for 7 min	20	1X Dream Taq Green Buffer, 2 mM $MgCl_2$, 200 µM of each dNTP, 0.4 µM of each primer, 0.5U Dream Taq DNA	[9]

						polymerase, 50 ng DNA template	
	FSY1	Seub_F3<br>Seub_R3	GTCCCTGTACCAATTTAATATTGCGC<br>TTTCACATCTCTTAGTCTTTTCCAGACG	95°C for 3 min, (95°C for 30 sec, 60°C for 30 sec, 72°C for 1 min)$_{35}$; 72°C for 7 min	20	1X Dream Taq Green Buffer, 2 mM $MgCl_2$, 200 µM of each dNTP, 0.4 µM of each primer, 0.5U Dream Taq DNA polymerase, 50 ng DNA template	[9]
Yeast genotyping	Microsatellite $(GTG)_5$	$(GTG)_5$	GTGGTGGTGGTGGTG	94 °C for 5 min; (94 °C for 15 s, 55 °C for 45 s, 72 °C for 1.30 min)$_{40}$; 72 °C for 4 min	20	1X Dream Taq Green Buffer, 3 mM $MgCl_2$, 200 µM of each dNTP, 0.6 µM of primer, 200 mM BSA, 0.5U Dream Taq DNA polymerase, 50 ng DNA template	[10]
	Random DNA	R3	ATGCAGCCAC	94°C for 4 min; (94°C for 25 sec, 42 C for 30 sec, 72 °C for 90 sec); 72°C for 5 min	20	1X Dream Taq Green Buffer, 3.0 mM $MgCl_2$, 200 µM of each dNTP, 1 µM of primer, 0.5 U/µL of Dream Taq DNA polymerase, 50 ng DNA template	[11]
	Transposable elements Ty2	d12<br>d21	TCAACAATGGAATCCCAAC<br>CATCTTAACACCGTATATGA	95°C for 5 min; (95°C for 30 s min, 46°C for 30 s, 72°C for 90 s)$_{35}$; 72°C for 10 min	25	1X Dream Taq Green Buffer, 2.5 mM $MgCl_2$, 200 µM of each dNTP, 1 µM of primer, 0.625U Dream Taq DNA polymerase, 50 ng DNA template	[12]
STA1	*STA1*	SD-5A<br>SD-6B	CAACTACGACTTCTGTCATA<br>GATGGTGACGCAATCACGA	95°C for 3 min; (95°C for 30 sec, 60°C for 30 sec, 72°C for 1 min)$_{35}$; 72°C for 7 min	20	1X Dream Taq Green Buffer, 2 mM $MgCl_2$, 200 µM of each dNTP, 0.4 µM of primer, 0.5U Dream Taq DNA	[13]

						polymerase, 50 ng DNA template	
	STA1 UAS2 promoter	STA1_UAS_Fw<br>STA1_UAS_Rv	CCTGGCTCAAATTAAACTTTCG<br>ACCACCAATAGGCAATAGAAA	95°C for 3 min; (95°C for 30 sec, 56°C for 30 sec, 72°C for 1 min)$_{35}$; 72°C for 7 min	20	1X Dream Taq Green Buffer, 2 mM $MgCl_2$, 200 μM of each dNTP, 0.4 μM of primer, 0.5U Dream Taq DNA polymerase, 50 ng DNA template	[14]
LAB identification	16S rRNA	27f<br><br>1490r	TCCATTTACTCGAGAGTTTGATCCTGG CTCAG<br>GGTTCCCCTAAGCTTACCTTGTTACG ACTTC	95 °C for 5 min; (95 °C for 1 min, 58 °C for 2.5 min, 72 °C for 2 min)$_{30}$; 72 °C for 5 min	40	1X TaKaRa *ExTaq* Buffer*, 2.0 μM $MgCl_2$, 200 μM of each dNTP, 0.2 μM of each primer, 1 U of TaKaRa *ExTaq* DNA polymerase, and 100 ng of template DNA	[15]
LAB genotyping	Microsatellite $(GTG)_5$	$(GTG)_5$	GTGGTGGTGGTGGTG	94 °C for 5 min; (94 °C for 15 s, 55 °C for 45 s, 72 °C for 1.30 min)$_{40}$; 72 °C for 4 min	20	1X Dream Taq Green Buffer, 3 mM $MgCl_2$, 200 μM of each dNTP, 0.6 μM of primer, 200 mM BSA, 0.5U Dream Taq DNA polymerase, 50 ng DNA template	[16]

Supplementary Table S2. *In silico* 16S-ARDRA profiles of the main beer LAB species.

Species	Accession number	*Tru1*I [1]	*Hha*I	*Hinf*I
Levilactobacillus brevis	HM058775.1	467, 259, 252, 194, 134, 104, 86, 47,26	367, 500, 571	274, 891, 85, 58
Fructilactobacillus lindneri	CP014907.1	109, 150, 44, 86, 48, 86, 252, 534	289, 528, 492	55, 976, 278
Lentilactobacillus buchneri	M58811	421, 252, 200, 194, 134, 123, 111, 86	400, 500, 580	785-540-135
Lactiactobacillus casei	D16551	464, 252, 239, 194, 134, 86, 81, 46, 26	580, 450, 400, 380, 210	1000, 145
Loigolactobacillus coryniformis	MF114100.1	465, 255, 206, 194, 156, 134, 86, 26	496, 528, 287	976, 277, 58
Secundilactobacillus malefermentans	NR_113822.1	505, 278, 86, 134, 44, 150, 111	1021, 287	493, 398, 274, 85, 58
Latilactobacillus curvatus	CP017124.1	593, 278, 161, 134, 86	528, 495, 287	976, 251, 58, 25
Pediococcus damnsosus	D87678	423, 254, 206, 200, 137, 134, 86, 44, 13	528, 507, 287	976, 263, 58, 25
Pediococcus acidilactici	M58833	278, 243, 205, 194, 134, 120, 104, 86, 79, 47, 36	500, 400, 320, 260	710, 380, 190
Pediococcus pentosaceus	AB362986.1	278, 269, 243, 137, 134, 120, 104, 86, 80, 47, 44, 36, 13	528, 507, 287	1000, 145
Pediococcus inopitalus	AJ271383	395, 252, 247, 137, 134, 121, 96, 86, 44, 26, 13	507, 528, 287	976, 263, 58, 25
Pediococcus parvulus	D88528	395, 252, 182, 178, 150, 134, 86, 44, 26	568, 526, 345	976, 263, 58, 25

[1] Fragments were in bp.

Supplementary Table S3. ITS RFLP-PCR analysis of yeast isolates from natural sour beer.

Isolation condition	Strain	Amplicon size[1]	Restriction profile		Yeast-ID best matching (%)	Pattern
			*Hae*III	*Hinf*I		
YPDA 28 °C	WY201, WY202, **WY203**, WY204, WY206, WY207, WY208, WY209, WY210, WY211, WY212, WY214, WY215, WY216, WY217, WY218, WY219, WY**220**	850	125, 170, 230, 325	110, 365, 375	*Saccharomyces cerevisiae* (100%)/*Saccharomyces coriocanus* (100%)/*Saccharomyces paradoxus* (100%)	A
	**WY205**, WY213	850	125, 230, 495	150, 365, 375	*Saccharomyces bayanus* (89%)/*Saccharomyces kudriavzevii* (89%)/*Saccharomyces pastorianus* (89%)/*Saccharomyces mikatae* (89%)	C
WL 28 °C	WY101, WY**104**, WY106, WY107, WY108, WY109, WY111, WY112, WY113, WY114, WY116, WY**117**, WY118, WY119, WY120, WY143, WY151	850	125, 170, 230, 325	110, 365, 375	*Saccharomyces cerevisiae* (100%)/*Saccharomyces coriocanus* (100%)/*Saccharomyces paradoxus* (100%)	A
	**WY115**	850	125, 230, 495	150, 365, 375	*Saccharomyces bayanus* (89%)/*Saccharomyces kudriavzevii* (89%)/*Saccharomyces pastorianus* (89%)/*Saccharomyces mikatae* (89%)	C
	**WY102**, WY103, WY105, WY110, WY121, WY**122**, WY141, WY152	485	48, 84, 336	203, 282	*Pichia membranifaciens* (73%)	B
	**WY59**, WY62	550	110, 400	95, 230	*Kluyveromyces blattae* (67%)	D
	**WY60**, WY61	490	100, 350	210, 280	*Dekkera bruxellensis* (83%)	E

[1] Amplicons and restriction fragments were in bp.

References

1. Saitou, N; Nei M. The neighbor-joining method: A new method for reconstructing phylogenetic trees. *Mol. Biol. Evol.*, **1987**, *4*, 406-425.
2. Tamura, K. Estimation of the number of nucleotide substitutions when there are strong transition-transversion and G + C-content biases. *Mol. Biol. Evol*. **1992**, *9*, 678-687.
3. Felsenstein, J. Confidence limits on phylogenies: An approach using the bootstrap. *Evolution*, **1985**, *39*, 783-791.
4. Kumar, S.; Stecher, G.; Li, M.; Knyaz, C.; Tamura, K. MEGA X: Molecular evolutionary genetics analysis across computing platforms. *Mol. Biol. Evol*. **2018**, *35*, 1547-1549
5. Letunic, I.; Bork, P. Interactive Tree of Life (iTOL) v4: Recent updates and new developments. *Nucleic Acids Res*. **2019**, *2*, 256–259.
6. Kurtzman, C.P., Fell, J.W., Boekhout, T.; Robert, V. Methods for isolation, phenotypic characterization and maintenance of yeasts. In *The yeasts*, 5st ed; Kurtzman, C.P.; Fell, J.W.; Boekhout, T., Eds.; Elsevier, Amsterdam, **2011**, pp. 87-110.
7. White, T.J.; Bruns, T.; Lee, S.; Taylor, J. Amplification and direct sequencing of fungal ribosomal RNA genes for phylogenetics. In *PCR Protocols: A Guide to Methods and Applications*; Innis, M.A., Gelfand, D.H., Sninsky, J.J., White, T.J., Eds.; Academic Press: San Diego, CA, USA, 1990; pp. 315–322.
8. Kurtzman, C.P.; Robnett, C.J. Identification and phylogeny of ascomycetous yeasts from analysis of nuclear large subunit (26S) ribosomal DNA partial sequences. *Antonie Leeuwenhoek*, **1998**, *73*, 331–371.
9. Pengelly, R.J.; Wheals, A.E. Rapid identification of *Saccharomyces eubayanus* and its hybrids. *FEMS Yeast Res*., **2013**, *13*, 156-161.
10. Dakal, T.C.; Solieri, L.; Giudici, P. Evaluation of fingerprinting techniques to assess genotype variation among *Zygosaccharomyces* strains. *Food Microbiol*. **2018**, *72*, 135-145.
11. Corte, L.; Lattanzi, M.; Buzzini, P.; Bolano, A.; Fatichenti, F.; Cardinali, G. Use of RAPD and killer toxin sensitivity in *Saccharomyces cerevisiae* strain typing. *J. Appl. Microbiol*. **2005**, *99*, 609-617.
12. Legras, J.-L.; Karst, F. Optimisation of interdelta analysis for *Saccharomyces cerevisiae* strain characterisation. *FEMS Microbiol. Lett.* **2003**, 221, 2249-2255.
13. Yamauchi, H.; Yamamoto, H.; Shibano, Y.; Amaya, N.; Saeki, T. Rapid methods for detecting *Saccharomyces diastaticus*, a beer spoilage yeast, using the polymerase chain reaction. *J. Am. Soc. Brew. Chem*. **1998**, *56*, 58–63.
14. Krogerus, K.; Magalhães, F.; Kuivanen, J. *et al.* A deletion in the *STA1* promoter determines maltotriose and starch utilization in *STA1+ Saccharomyces cerevisiae* strains. *Appl Microbiol Biotechnol*. **2019**, *103*, 7597–7615.
15. Lane, D. J. 16S/23S rRNA sequencing. In *Nucleic Acid Techniques in Bacterial Systematics*; Stackebrandt, E.; Goodfellow, M., Eds; John Wiley & Sons: New York, NY, USA, **1991**; pp. 115–175.
16. Solieri, L.; Bianchi, A.; Giudici, P. Inventory of non-starter lactic acid bacteria from ripened Parmigiano-Reggiano cheese as assessed by a culture dependent multiphasic approach. *Syst. Appl. Microbiol*. **2012**, *35*, 270–277.
